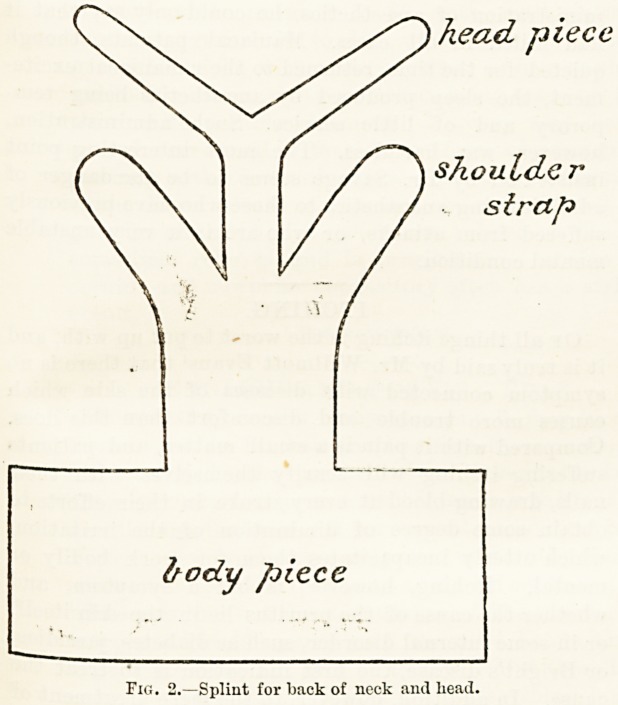# Some Plaster of Paris Splints

**Published:** 1899-11-18

**Authors:** L. A. Bidwell

**Affiliations:** Senior Assistant Surgeon to the Hospital, Dean of the Postgraduate College.


### Hospital Clinics and Medical Progress.

### SOME PLASTER OF PARIS SPLINTS.

A Postgraduate Lecture, delivered at the West London Hospital by L. A. Bidwell, F.R.C.S., Senior Assistant Surgeon to the Hospital, Dean of the Postgraduate College.

(*Continued from page* 92.)

The next splint to which I wish to draw your attention is that used in the treatment of fracture of the femur; it is commonly called a half trouser, and is made after the fashion of Croft's splints. Before describing the splint I should like to impress upon you that it is practically impossible to apply this or any other splint with any hope of preventing shortening unless a general anæsthetic be given. Under ether the muscular contraction is overcome, and by the time the patient comes to the splint has set, and so any return of the shortening is prevented. This splint, of course, fixes both the knee and the hip. The measurements required are: (1) The circumferences just below the knee, about the knee, and at the upper part of the thigh; then the length from two fingers' breadth below the costal margin to the middle of the calf, and from the middle of the crest of the ilium to the opposite anterior superior iliac spine. This splint should be applied, as I said, under ether; a weight extension should be attached to the leg, and a long outside splint put on over the plaster of Paris and allowed to remain for the first 10 days, after which it may be removed and the patient may be allowed to get up on crutches, wearing only the plaster splint.

Fig. 2.—Splint for back of neck and head.

The last splint which I will show is one which is designed to fix the head and back of the neck in a case of acute spinal caries or of fracture of the cervical spine. It is very useful, as it allows the patient to get about while a more permanent apparatus is being made. It is made after the pattern shown in Fig. 2; the top strip grips the head, and the two ends meet over the forehead, the middle bands are moulded over the shoulders, and the lowest part of the splint goes two-thirds round the chest below the axillæ, the back of the splint is moulded to the back of the cervical and upper dorsal spine. It is best to cut out a paper pattern, which must be done on a lengthways fold of paper, so that the two sides shall be symmetrical; two pieces of flannel are cut and prepared in a similar manner to a Croft's splint, and the splint is applied directly to the back, as the inner side is free from plaster. The chest and shoulder flaps are moulded on, and fixed with a figure-of-8 bandage round the shoulders and chest, so that the two head-pieces will be found to overlap over the forehead, and are secured by a bandage till they set; another temporary bandage moulds the back of the splint to the cervical spine. In order to make this splint stronger it is often advisable to put a small wooden lath, or, better still, a piece of iron band such as is used in packing-cases, between the two layers of the flannel opposite the neck; the chin, too, can be supported by a piece of bandage passed beneath the forehead part of the splint on each side above and beneath the chin below.

In conclusion, I would point out that these splints do not require the surgeon to keep a stock of apparatus—only plaster of Paris being needed. If no house-flannel can be obtained, an old blanket will answer the purpose excellently; and although some assistance is necessary, it need not be skilled, since, except in the case of a fracture of the femur, any friend of the patient can hold the splint in position while setting.

## Figures and Tables

**Fig. 2. f1:**